# Successful Management of Mixed Mycosis in HIV-Negative Patients With Different Immune Status: A Case Series Report

**DOI:** 10.3389/fcimb.2022.851891

**Published:** 2022-03-04

**Authors:** Yangqing Zhan, Chun Lu, Shaoqiang Li, Jin Zhao, Zhengtu Li, Yingying Gu, Feng Ye

**Affiliations:** ^1^ Department of Pulmonary and Critical Care Medicine, The First Affiliated Hospital of Guangzhou Medical University, Guangzhou, China; ^2^ Guangzhou Institute of Respiratory Health, State Key Laboratory of Respiratory Disease, National Clinical Research Center for Respiratory Disease, Guangzhou, China; ^3^ The First Affiliated Hospital of Guangzhou Medical University, Guangzhou Medical University, Guangzhou, China

**Keywords:** immunocompetent, immunocompromised, *Mucor*, *Aspergillus*, *Cryptococcus*, *Talaromyces marneffei*

## Abstract

**Objective:**

The limited information available on mixed mycosis involving the lungs makes the understanding of mixed fungal diseases insufficient and affects prognosis. Our study aims to improve understanding by exploring experience in the successful management of mixed fungal infections.

**Methods:**

Patients who had two types of mycosis involving the lung at the same disease course were retrospectively enrolled.

**Results:**

Between September 2011 and December 2019, 17 patients with proven mixed mycosis were enrolled. Four patients were immunocompromised, with one case each of lung transplantation, corticosteroid treatment, STAT3 hyper-IgE syndrome, and anti-IFN-γ autoantibody-associated immunodeficiency syndrome. Among 13 patients who were not immunocompromised, 9 had type 2 diabetes mellitus. Eight cases were coinfection with *Mucor* and *Aspergillus*, 4 cases were *Cryptococcus* and *Aspergillus*, 2 cases were *Talaromyces marneffei* and *Cryptococcus*, 2 cases were *Talaromyces marneffei* and *Aspergillus*, and 1 case was *Candida* and *Aspergillus*. Seven patients were diagnosed with mixed pulmonary mycosis at almost the same time. Among the remaining 10 patients, the initial treatment was ineffective in four cases, and six patients showed a partial response to the initial antifungal treatment, but the original fungal lesions became re-enlarged. Three patients were admitted to the intensive care unit during hospitalization, and one patient died. Another *Mucor* coinfection patient died due to treatment refusal.

**Conclusion:**

Mixed mycosis involving the lungs is not uncommon in patients without apparent immune deficiency diseases. During the management of mycosis, we recommend keeping mixed mycosis in mind for patients with a poor response to initial antifungal treatment, even in immunocompetent populations, and identifying the cause of illness through a rigorous procedure.

## Introduction

Fungi can cause serious infections worldwide, with the number of serious cases reaching 150 million (Bongomin et al., 2017). Immunocompromised hosts, such as those with organ transplantation, hematopoietic stem cell transplantation, malignant tumors receiving radiotherapy and chemotherapy, and autoimmune disease receiving immunosuppressive therapy, are at risk of invasive pulmonary mycosis, which may lead to poor prognosis. On the basis of discharge diagnoses, the incidence of invasive fungal infections in France was 5.9/100 000 cases/year, with a mortality of 27.6%, both of which increased during the observation period (2001–2010) (von Lilienfeld-Toal et al., 2019). While, the proportion of pulmonary fungal infection increased annually from 26.5 per 1000 inpatients in 2013 to 42.6 in 2019 and from 3.07 per 1000 outpatients in 2013 to 8.48 in 2019 in Guangzhou, China (Li et al., 2021). The most common pulmonary mycoses are *Aspergillus, Candida, Cryptococcus* species, and *Pneumocystis jirovecii* (Bongomin et al., 2017; Azoulay et al., 2019; Zhan et al., 2021). Mixed fungal infections have also been reported (Wang et al., 2018; Awari et al., 2020); however, their characteristics have not yet been described in detail, and most are in immunocompromised hosts.

Existing data show that pulmonary fungal disease is not limited to immunosuppressed hosts. Reports of pulmonary fungal diseases in immunocompetent hosts are gradually increasing, such as cryptococcosis, *Talaromycosis marneffei* infection, and aspergillosis, although the latter is one of the common infections in primary immunodeficiency diseases such as chronic granulomatous diseases (Mortaz et al., 2019; Abd Elaziz et al., 2020). In China, most patients with cryptococcosis do not have an immunocompromised status (Ye et al., 2012; Cheng et al., 2021). *Talaromycosis marneffei* infection, an endemic fungal disease in Asian populations, also occurs in HIV-negative immunocompetent populations (Lee et al., 2019; Pan et al., 2020). Although some patients have been confirmed to have nonclassical immunodeficiency, such as hyper-IgE syndrome and anti-IFN-γ autoantibody-associated immunodeficiency syndrome, most patients do not have clear immunodeficiency or hypofunction (Lee et al., 2019; Pan et al., 2020). Due to the lack of specificity in the clinical manifestations of pulmonary mycosis in immunocompetent hosts, mycosis is often not the first consideration during clinical diagnosis and treatment. Incorrect diagnosis often delays treatment, which may affect the prognosis.

Diagnosis and treatment are even more difficult in mixed fungal disease because of unsatisfied positivity in culture and pathology. The need for timely inoculation, destruction of hyphae by tissue grinding, and empiric use of antifungal drugs is one of the reasons for negative culture. The morphological similarity of mold makes pathological diagnosis dependent on experienced technicians. Awari et al. and Wang et al. reported some cases of mixed mycoses of *Aspergillus* and *Cryptococcus* in both immunocompromised and immunocompetent patients (Wang et al., 2018; Awari et al., 2020). Coinfection with *Talaromyces marneffei* and *Cryptococcus neoformans* has also been reported in a non-HIV patient (He et al., 2020). However, the characteristics of fungal coinfection are still undefined. Here, we retrospectively analyzed a group of successively managed mixed mycoses to explore the clinical characteristics and main points of diagnosis and treatment of mixed mycoses.

## Methods

This was a retrospective study conducted at the First Affiliated Hospital of Guangzhou Medical University. Between Jan 1, 2011 and Dec 31, 2019, patients who were hospitalized in the Department of Pulmonary and Critical Care Medicine with a diagnosis of mixed mycosis that involved pulmonary were searched from the electronic medical record database and enrolled as the study group. This study was approved by the ethics committee of the First Affiliated Hospital of Guangzhou Medical University (2018-119). Because this is a retrospective study, informed consent was not required.

### Study Population

Inclusion criteria were the following: I. patients who had two types of fungal infections involving the lung at the same disease course; II. patients who had follow-up data in the outpatient medical records database; and III. patients who were HIV negative.

Mycosis was diagnosed based on the European Organization for Research and Treatment of Cancer and the Mycoses Study Group Education and Research Consortium (EORTC/MSGERC), which is a graded diagnostic standard, including the classifications of “proven,” “probable,” and “possible” invasive fungal disease (IFD) (Donnelly et al., 2019). The present study included only patients with proven or probable IFD. The diagnostic criteria for proven IFD included a specimen obtained by needle aspiration or biopsy showing the unique morphology of the fungus by histopathology, cytopathology or direct microscopic examination; a specimen obtained by a sterile procedure from a normally sterile and clinically or radiologically abnormal site consistent with an infectious disease process, recovering a hyaline or pigmented mold or yeast by culture; a positive blood culture for mold (*Aspergillus* was excluded) or yeast; or a positive test for cryptococcal antigen in cerebrospinal fluid or blood. The diagnosis of probable IFD should meet all three criteria, including host factors, clinical features and mycological evidence, with details in the referenced guideline (Donnelly et al., 2019).

Exclusion criteria included the following: I. two fungal infections did not occur at the same disease course, and the first mycosis was cured before the onset of the second mycosis; II. only one fungal infection was diagnosed; and III. there was incomplete information.

### Data Collection

Clinical features of enrolled patients were collected, including medical history, underlying diseases, use of corticosteroids and/or immunosuppressants before the onset of mycosis, clinical symptoms and signs, imaging and laboratory examination data, types of culture specimens, diagnosis procedure, treatment, and outcome. If the patient went to the outpatient clinic, the follow-up and results within 1 year were also collected from the database.

### Statistics

SPSS 25.0 software was used for data processing of the results of this study. Because the treatment drugs and prognosis of mucormycosis are different from those of other fungal infections, comparisons were made between *Mucor* mixed infection and non-*Mucor* mixed infection cases. All *P* values are two-sided tests, and *P <*0.05 means that the difference is statistically significant. Independent sample t-tests were used for continuous variables, and Fisher’s exact test and χ2 test were used for categorical variables.

## Results

### General Characteristics

Between September 2011 and December 2019, 17 cases of mixed fungal infection were confirmed in our hospital. The average age was 50.2 ± 16.9 years, ranging from 21 to 76, with 12 (70.6%) males. Four patients were immunocompromised, with one patient receiving bilateral lung transplantation for destructive pneumonophthisis, one patient receiving long-term corticosteroid therapy for nephrotic syndrome and hypoalbuminemia, one case of STAT3 hyper-IgE syndrome and one case of anti-IFN-γ autoantibody-associated immunodeficiency syndrome (**Table 1**). Among the 13 patients who did not have apparent immune deficiency, 10 had underlying diseases, including hypertension, diabetes mellites and hyperthyroidism (**Table 1**).

**Table 1:** Clinical course of 17 patients with mixed mycosis.

Case No.	Sex	Age	Smoking history	Comorbidity	Diagnosis of mycosis	Classification of IFD by EORTC/MSG	Host factors	Clinical features	Mycological evidence	Biopsy	Clinical course of illness	Outcome
1	Female	67	No	Congenital lung cyst, hyperthyroidism	Chronic pulmonary aspergillosis and pulmonary cryptococcosis	Proven	NA	Lesions	Negative	*Aspergillus* and C*ryptococcus*	Diagnosis of mixed mycosis was made after surgery and itraconazole was prescribed	Survival
2	Female	50	No	Type 2 diabetes mellitus, hypertension	Disseminated cryptococcosis (lung and lymph nodes) and pulmonary aspergillosis	Proven	NA	Consolidation	Negative	*Aspergillus* and C*ryptococcus*	Disseminated cryptococcosis (lung and lymph nodes) was diagnosed at first, with poor response to 5 months of treatment with fluconazole. Surgery was performed after intolerance to amphotericin B and a poor response to voriconazole for 1 month. Biopsy showed coinfection of *Aspergillus* and *Cryptococcus*.	Survival
3	Female	66	No	Type 2 diabetes mellitus	Invasive pulmonary aspergillosis and pulmonary mucormycosis	Proven	NA	Lesions and cavity	Negative	*Aspergillus* and *Mucor*	Voriconazole was prescribed after consideration of invasive fungal disease in the lung and poor status of the patient. The lesion showed a good response, but was re-enlarged 2 months after treatment. Biopsy *via* bronchoscopy confirmed the mixed mycosis. There was a good response after posaconazole treatment.	Survival
4	Male	57	Yes	Type 2 diabetes mellitus	Invasive pulmonary aspergillosis and pulmonary mucormycosis	Proven	NA	Consolidation and cavity	Negative	*Aspergillus* and *Mucor*	Mixed infection was diagnosed concurrently, with a good response to amphotericin B liposome treatment.	Survival
5	Male	24	No	Hyper-IgE syndrome and bronchiectasis	Disseminated *Talaromyces marneffei infection* (lung, fungemia) and invasive pulmonary aspergillosis	Proven	STAT3 deficiency	Cavity	Negative	*Aspergillus* and *Talaromyces marneffei*	Disseminated *Talaromyces marneffei* infection was diagnosed at first and was treated with itraconazole because of intolerance to amphotericin B due to vomiting. Four months later, surgical resection was performed for new onset of hemoptysis and gradual enlargement of original lesions in chest CT scans.	Recurrence of *Talaromyces marneffei* infection during antifungal treatment
6	Female	61	No	Type 2 diabetes mellitus	Invasive pulmonary aspergillosis and pulmonary mucormycosis	Proven	NA	Consolidation and cavity	*Aspergillus fumigatus* by culture	*Aspergillus* and *Mucor*	Mixed infection was diagnosed concurrently. But the patient was severely ill with poor condition and refused treatment.	Death
7	Male	66	Yes	Lung transplantation, hypertension	Invasive pulmonary aspergillosis and pulmonary candidiasis	Proven	Lung transplantation	Consolidation	*Aspergillus fumigatus* by culture	*Aspergillus* and *Candida*	Invasive pulmonary aspergillosis was diagnosed after a positive result in sputum culture. The patient showed a poor response to voriconazole. Mixed mycosis was diagnosed after bronchoscopy, and amphotericin B was given subsequently. However, there was a poor response.	Death
8	Male	46	Yes	Type 2 diabetes mellitus	Invasive pulmonary aspergillosis and pulmonary mucormycosis	Proven	NA	Lesions, consolidation and cavity	Negative	*Aspergillus* and *Mucor*	Pulmonary mycosis was considered after bronchoscopy biopsy at another hospital. There was no change in the lesion after 2 months of voriconazole. Mixed mycosis was diagnosed after review of the biopsy and showed good response to amphotericin B.	Survival
9	Male	25	Yes	Type 2 diabetes mellitus	Invasive pulmonary aspergillosis and pulmonary mucormycosis	Proven	NA	Lesions, consolidation and cavity	Negative	*Aspergillus* and *Mucor*	Mixed infection was diagnosed concurrently, with a good response to amphotericin B liposome treatment.	Survival
10	Male	40	No	None	Pulmonary cryptococcosis and chronic pulmonary aspergillosis	Proven	NA	Consolidation and cavity	Negative	*Cryptococcus* and *Aspergillus*	Pulmonary cryptococcosis was confirmed at first, with a poor response to 8 months of treatment with fluconazole. Mixed mycosis was diagnosed after review of the biopsy and showed good response to voriconazole with therapeutic drug monitoring.	Survival
11	Male	21	No	Anti-IFN-γ autoantibody-associated immunodeficiency syndrome	Disseminated *Talaromyces marneffei* infection (lung, lymph nodes, bone) and invasive pulmonary aspergillosis	Proven	IFN-γ antibody deficiency	Consolidation	Serum galactomannan antigen > 1.0	*Talaromyces marneffei*	Disseminated *Talaromyces marneffei* was diagnosed at first and showed a good response to amphotericin B and itraconazole successively. There was new occurrence of lesions and re-enlargement of original lesions 9 months later, and mixed mycosis was diagnosed, with a good response to successive amphotericin B and voriconazole.	Recurrence of *Talaromyces marneffei* infection during antifungal treatment
12	Male	41	No	Nephrotic syndrome and hypertension	Disseminated cryptococcosis (lung and CNS) and pulmonary *Talaromyces marneffei* infection	Proven	Corticosteroid treatment for nephrotic syndrome	Lesions and cavity	Cryptococcal antigen in blood and cerebrospinal fluid	*Talaromyces marneffei* and *Cryptococcus*	Pulmonary cryptococcosis was confirmed at first, with a poor response to 1 month of treatment with fluconazole. Mixed infection was diagnosed after review of the biopsy, with a good response to successive amphotericin B liposome plus flucytosine and voriconazole.	Survival
13	Female	76	No	Type 2 diabetes mellitus	Pulmonary mucormycosis and invasive pulmonary aspergillosis	Proven	NA	Lesions, consolidation and cavity	Negative	*Aspergillus* and *Mucor*	Pulmonary mucormycosis was diagnosed at first and showed a good response to posaconazole because of intolerance to amphotericin B due to heart arrest. There was new occurrence of hemoptysis and re-enlargement of original lesions 5 months later, and invasive pulmonary aspergillus was diagnosed, with a good response to voriconazole.	Survival
14	Male	37	No	None	Pulmonary cryptococcosis and pulmonary aspergillosis	Proven	NA	Lesions	Cryptococcal antigen in blood	*Aspergillus* and Cryptococcus	Pulmonary cryptococcosis was diagnosed at first and showed a good response to fluconazole. There was re-enlargement of original lesions 14 months later, and mixed mycosis was diagnosed, with a good response to voriconazole.	Survival
15	Male	48	No	Type 2 diabetes mellitus and diabetic nephropathy	Pulmonary mucormycosis and pulmonary aspergillosis	Proven	NA	Lesions and consolidation	Negative	*Aspergillus* and *Mucor*	Pulmonary mucormycosis was diagnosed at first and showed a good response to successive amphotericin B plus posaconazole and posaconazole. There was enlargement of original lesions 8 months later, and mixed mycosis was diagnosed, with a good response to voriconazole.	Survival
16	Male	62	No	Chronic renal failure	Disseminated *Talaromyces marneffei* infection (lung and skin) and pulmonary cryptococcosis	Proven	NA	Lesions	Cryptococcal antigen in blood and *Talaromyces marneffei* by culture	Negative	Mixed infection was diagnosed concurrently, with a good response to successive amphotericin B and voriconazole.	Survival
17	Male	66	Yes	Type 2 diabetes mellitus and hypertension	Invasive pulmonary aspergillosis and pulmonary mucormycosis	Proven	NA	Lesions and cavity	Negative	*Aspergillus* and *Mucor*	Mixed infection was diagnosed concurrently, with a good response to successive amphotericin B plus posaconazole.	Survival

Among all 17 patients, 8 cases were coinfection with *Mucor* and *Aspergillus*, 4 cases with *Cryptococcus* and *Aspergillus*, 2 cases with *Talaromyces marneffei* and *Cryptococcus*, 2 cases with *Talaromyces marneffei* and *Aspergillus*, and 1 case with *Candida* and *Aspergillus*. The lesions of all patients with mucormycosis were confined to the lungs, but some patients with *Talaromyces marneffei* infection and cryptococcosis had involvement of extra-pulmonary organs, including bone, skin, lymph nodes, and/or central nervous system (CNS) (**Table 1**).

### Clinical Symptoms, Signs and Laboratory Tests

Cough, expectoration and fever were the most common symptoms. More than 40% of patients had hemoptysis or shortness of breath. Only 3 patients (17.6%) had an elevated white blood cell count. There were no decreased immunoglobulin G/M/A levels or CD4+ or CD8+ T lymphocyte cell counts in patients without apparent immune deficiency diseases (**Table 2**).

**Table 2:** Clinical characteristics of 17 patients with mixed fungi.

Clinical features	N(%)
Fever	11 (64.7%)
Cough	15 (88.2%)
Expectoration	15 (88.2%)
Hemoptysis	8 (47.1%)
Shortness of breath	7 (41.2%)
Chest pain	4 (23.5%)
Weight loss	2 (11.8%)
Wet rales	4 (23.5%)
White blood cell count>10×10^9^/L	3 (17.6%)
White blood cell count 4-10×10^9^/L	14 (82.4%)
Hemoglobin (g/L, mean ± SD)	108.5 ± 21.9
Lactic dehydrogenase (U/L, mean ± SD)	193.6 ± 62.2
Aspartate aminotransferase (U/L, mean ± SD)	29.3 ± 32.1
Alanine aminotransferase (U/L, mean ± SD)	20.9 ± 14.9
Albumin (g/L, mean ± SD)	37.4 ± 7.2
C-reactive protein (g/L, mean ± SD)	6.7 ± 7.5
PCT (ng/L, mean ± SD)	0.1 ± 0.2
CD4+ T lymphocytes (cells/µl)	780 ± 389
CD8+ T lymphocytes (cells/µl)	822 ± 475

Data are shown as numbers (%), unless otherwise specified.

### Mycological Results and Biopsy

The culture was positive for *Aspergillus fumigatus* in case 6 and case 7 and for *Talaromyces marneffei* in case 11. A galactomannan antigen test was positive in case 11. A cryptococcal antigen test was positive in case 14 and case 16. Biopsy was positive in all patients but case 16 (**Table 1**).

### Manifestations of CT Images

**Figure 1:**
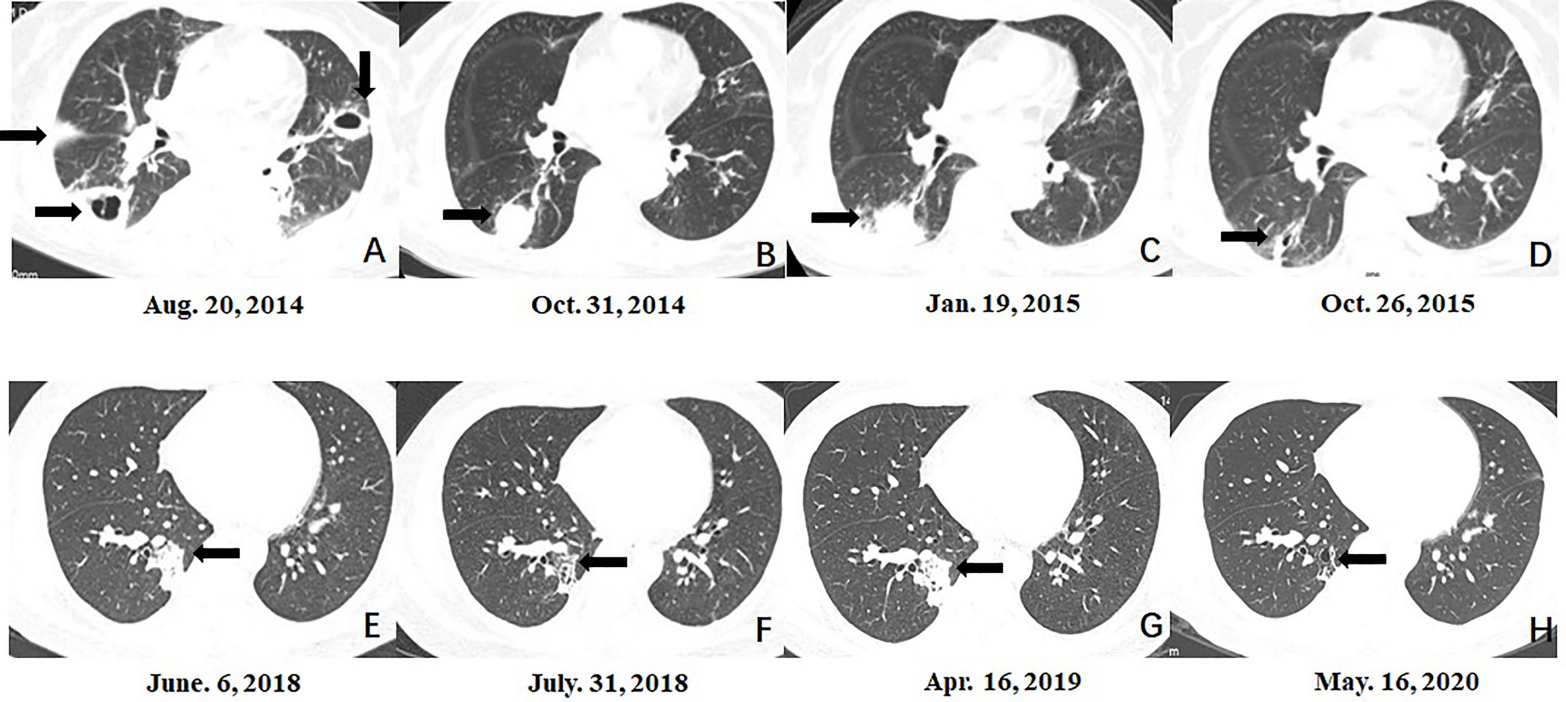
Serial morphologic changes on chest CT of patients with mixed mycosis. **(A–D)** CT images in case 3 showed that compared to the lesion at the time of pulmonary aspergillus diagnosis **(A)**, the lesion in the right lower lobe was smaller at 3 months after voriconazole treatment **(B)**, re-enlarged 5 months after voriconazole treatment **(C)**, and almost disappeared at 9 months after the diagnosis of mixed mycosis and posaconazole treatment **(D)**. **(E–H)** CT images in case 14 showed that compared to the lesion at the time of pulmonary cryptococcosis diagnosis **(E)**, the lesion in the right lower lobe was smaller 8 weeks after fluconazole treatment **(F)**, re-enlarged 10 months after fluconazole treatment **(G)**, and almost disappeared at 1 year after the diagnosis of mixed mycosis and voriconazole treatment **(H)**.

All 17 patients underwent chest CT examination. The most common features of CT images were mediastinal lymph node enlargement (12 cases, 70.6%), cavity (10 cases, 58.5%), effusion or consolidation (9 cases, 52.9%), multiple nodules (7 cases, 41.2%) and pleural effusion (7 cases, 41.2%, **Table 3** and **Figure 1**). However, none of the 17 cases showed classical features of lung mycosis, such as halo signs and crescent signs. There was also no vascular truncation sign or anti-halo sign. Lesions were less commonly located in the lingual lobe than in other lobes (**Table 3**).

**Table 3:** Manifestations of lung CT images and distribution of lesions in patients with mixed fungi.

Imaging feature or location of lesions	N (%)
Lobulation	2 (11.8%)
Cavity	10 (58.8%)
Necrosis	1 (5.9%)
Single nodule/mass	1 (5.9%)
Multiple nodules/masses	7 (41.2%)
Effusion or consolidation	9 (52.9%)
Lymph node enlargement	12 (70.6%)
Thickening of the pleura	1 (5.9%)
Pleural effusion	7 (41.2%)
Left upper lobe	8 (47.1%)
Left lingual lobe	5 (29.4%)
Left lower lobe	9 (52.9%)
Right upper lobe	11 (64.7%)
Right middle lobe	7 (41.2%)
Right lower lobe	10 (58.8%)

### Clinical Course, Treatment, and Prognosis

**Figure 2:**
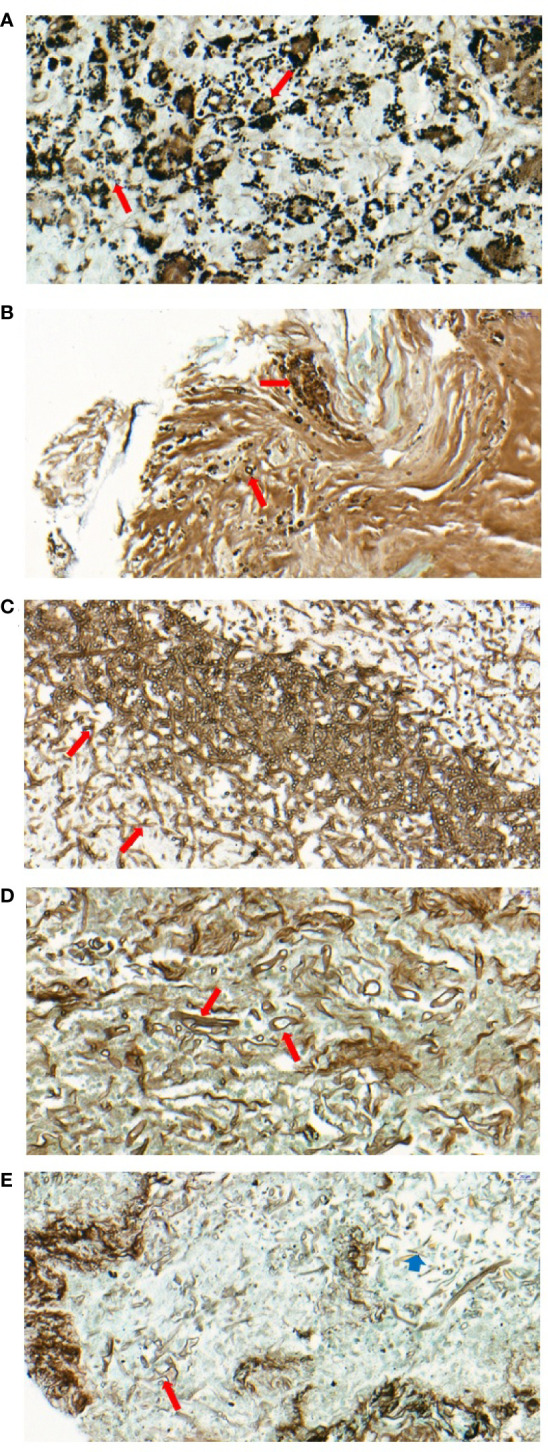
Pathology of patients with mixed mycosis. **(A)** Multiple spores can be seen in the cytoplasm of the multinucleated giant cells in the granulomatous nodules of lymphoid tissue in case 5. GMS staining (400x) showed that the fungi grew in clusters, with separation within the fungus and a small black spot in the center, which was considered *Talaromyces marneffei* (long red arrow). **(B)** Focal necrosis containing round or semilunar spores can be seen under the bronchial mucosal tissue with GMS staining (400x), which was considered to be *Cryptococcus* (long red arrow) in case 10. **(C, D)** Two forms of hyphae and spores can be seen in the necrotic foci of lung tissue by GMS staining (400x) in case 15. Fungi with the same thickness of hyphae and acute angle of branches were considered *Aspergillus* (long red arrow) **(C)**, fungi with different sizes of hyphae and strange shapes of branches were considered *Mucor* (long red arrow) **(D)**. **(E)** Two forms of hyphae and spores can be seen in the necrotic foci of lung tissue by GMS staining (400x) in case 17. Fungi with different sizes of hyphae, strange shapes of branches and thickened capsules were considered *Mucor* (long red arrow); fungi with the same thickness of hyphae and acute angle of branches were considered *Aspergillus* (short blue arrow).

Seven patients were diagnosed with mixed pulmonary mycosis at almost the same disease course. Among the remaining 10 patients, the initial treatment was ineffective in three cases (cases 8, 10, and 12; fluconazole in two cases and voriconazole in 1 case), and the patients were diagnosed with mixed mycoses after review of the original biopsy from the hospital to which they were first admitted (**Figure 2**). They showed good response to adjusted treatment. The original fluconazole, second-line amphotericin B and voriconazole were ineffective in case 2. She was diagnosed with mixed mycosis after surgical resection. In six cases (cases 3, 5, 11, 13, 14, and 15), mixed mycosis diagnosis was made after bronchoscopy biopsy of re-enlarged original fungal lesions, which showed a partial response to initial antifungal treatment (fluconazole in 1 case, voriconazole in 1 case, itraconazole in 2 cases and posaconazole in 2 cases) (**Figure 1**). Among the 10 cases of mixed mycoses that were not diagnosed at the same time, initial antifungal drugs in 6 cases had no activity on the second fungus.

Three patients were admitted to the intensive care unit during hospitalization. However, patient 7 died even after treatment in the intensive care unit, and patient 6 died due to treatment refusal. Patient 2 showed a poor response to antifungal treatment, and pneumonectomy was conducted. Patients 5 and 11 suffered from a recurrence of disseminated *Talaromyces marneffei* infection even under the course of antifungal treatment. The remaining patient showed improvement in the CT scan with no recurrence.

### Comparisons of Patients With *Mucor* Coinfection and Without *Mucor* Coinfection

**Figure 3:**
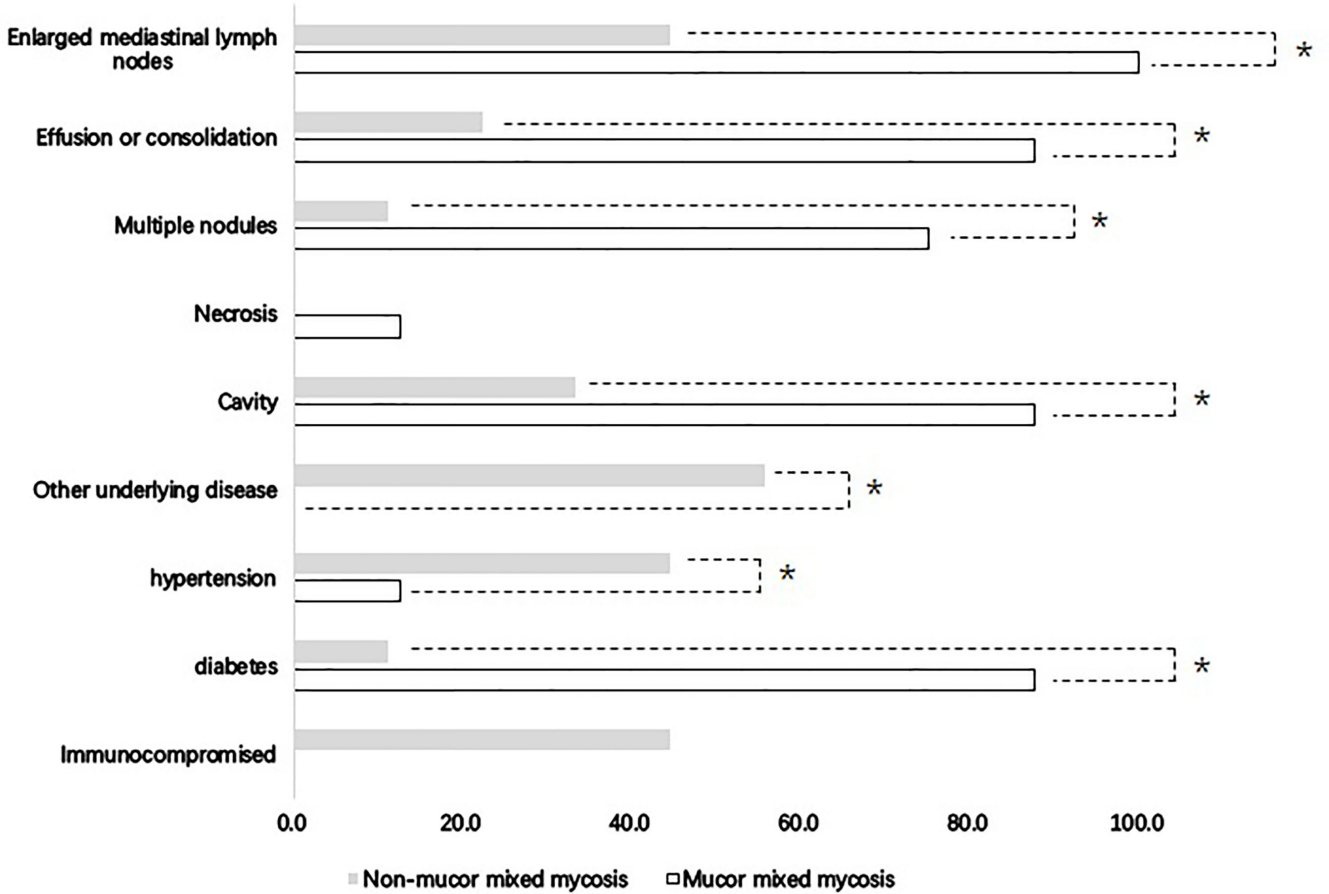
Clinical features of mixed mycosis patients with and without *Mucor* infection. *Comparisons between *Mucor* mixed mycosis and non-*Mucor* mixed mycosis showed significance (P < 0.05).

Because voriconazole is ineffective for *Mucor*, which has a poor prognosis, comparisons were made between patients with *Mucor* and without *Mucor* coinfection to discriminate the characteristics between these two groups. There were 8 cases with *Mucor* coinfection and 9 cases without *Mucor* coinfection. Patients with *Mucor* coinfection seemed to be older than patients without *Mucor* coinfection; however, the difference was not significant (55 ± 16 years vs. 45 ± 16 years, *P*=0.22). Diabetes mellitus was more common in patients with *Mucor* coinfection than in patients without *Mucor* coinfection (**Figure 3**). Patients with mixed *Mucor* infection had lower hemoglobin levels than patients without mixed *Mucor* infection (97 ± 15 g/L vs. 118 ± 23 g/L, *P*=0.037). Cavity, multiple nodule effusion/consolidation and enlarged mediastinal lymph nodes were more common in *Mucor* mixed infection than in non-*Mucor* mixed infection (**Figure 3**). Mortality was similar in the two groups (12.5% vs. 11.1%, *P*>0.05).

## Discussion

In the past, it was believed that pulmonary mycosis occurred only in immunosuppressed hosts, with *Candida*, *Aspergillus* and *Cryptococcus* as the most common opportunistic fungi (Pfaller and Diekema, 2004), but increasing evidence shows that pulmonary mycosis in patients without apparent immune deficiency diseases is not uncommon, especially pulmonary cryptococcosis, which mainly occurs in immunocompetent hosts in mainland China. In our study, patients without apparent immunocompromising disease or immunosuppressive treatment accounted for an extremely large proportion.

It should be noted that only 2 cases in this study were in classical immunocompromised hosts, and one of them developed a mixed infection with *Talaromyces marneffei* in the present study. Another two cases of *Talaromyces marneffei* mixed infection were confirmed to have atypical types of immunodeficiency after the occurrence of *Talaromyces marneffei* infection, one with STAT3 hyper-IgE syndrome and the other with anti-IFN-γ autoantibody-associated immunodeficiency syndrome. The former was originally characterized by recurrent cold staphylococcal abscesses, pneumonia, eczema, hyperextensibility, and extreme elevation of IgE levels (Holland et al., 2007). The latter is associated with severe disseminated nontuberculous mycobacterial infections and other disseminated opportunistic infections (*Salmonella*, *Histoplasma*, and *Cryptococcus*) in previously healthy adults (Hong et al., 2020). However, recent studies have shown that these populations are also susceptible to infection with *Talaromyces marneffei*, which was previously a significant infectious complication in HIV/AIDS patients but has now increased in incidence in patients with other immune defects (Cao et al., 2019; Lee et al., 2019). Therefore, it is necessary to identify the potential cause of immunodeficiency in cases of *Talaromyces marneffei* infection, and people with unapparent immunodeficiency need to pay attention to the possibility of mycoses, especially those caused by *Talaromyces marneffei*.

In the present study, all but one patient infected by *Mucor* had type 2 diabetes mellites. The incidence of diabetes mellites was more common in the mucormycosis group than in the non-mucormycosis group. This result was consistent with the literature indicating that diabetes mellites, with or without ketoacidosis, is one of the risk factors for *Mucor* infection (Cornely et al., 2019). Although the mechanism of immunodeficiency in diabetic patients is still unclear and needs in-depth research, if diabetic patients with pulmonary mycosis do not have a good response to the existing antifungal treatment that does not cover *Mucor*, attention should be given to the possibility of *Mucor* coinfection. Additionally, there were 3 non-immunocompromised hosts with mixed fungal diseases and no underlying diseases. It is valuable information for clinicians because not only single pulmonary fungal disease but also mixed fungal diseases can occur in patients without apparent immune deficiency diseases. According to our experience, during the follow-up, if the patients with proven pulmonary fungal disease showed a poor response to antifungal treatment, the existence of another fungal disease needs to be considered even in patients without apparent immune deficiency diseases.

Regardless of the immune status, among the single IFDs, *Aspergillus* and *Cryptococcus* are the most common pathogens, while *Mucor* and *Talaromyces marneffei* are relatively rare (Letourneau et al., 2014; Yan et al., 2016; Cao et al., 2019). *Pneumocystis jiroveci* is also one of the most common pathogens in immunosuppressed hosts (Azoulay et al., 2019). However, the mixed mycoses reported in the literature are mainly mixed infections of *Aspergillus* and *Cryptococcus* (Lin et al., 2006; Enoki et al., 2012; Wang et al., 2018; Awari et al., 2020). There have also been reports of *Aspergillus* and *Mucor* coinfection in the lung or outside the lungs (Bergantim et al., 2013; Mahadevaiah et al., 2013; Webb et al., 2013). Most *Cryptococcus* and *Talaromyces marneffei* coinfections have been reported in HIV patients (Le et al., 2010; Groll et al., 2021). However, in the present study, mixed fungal infections with *Aspergillus* and *Mucor* were the most common types, followed by *Aspergillus* and *Cryptococcus* coinfection, and coinfection with *Talaromyces marneffei* and other fungi was not uncommon in HIV-negative patients. This information is valuable for clinicians. When the initial antifungal treatment is not effective and mixed fungal diseases are considered, the possibility of *Mucor* should be considered, especially in patients who have diabetes mellites. Therefore, fungus coinfection is worthy of consideration.

The symptoms, signs and imaging findings of mixed fungal lung infection lack specificity. There is no halo sign or crescent sign, which is not consistent with the imaging features of previous invasive pulmonary aspergillosis with a “halo sign at the early stage and crescent sign at the later stage” (Park et al., 2010). Classical chest CT images of *Mucor* infection, such as reversed halo signs and vascular occlusion, were also lacking in the present *Mucor* patients. Although *Mucor* patients more commonly showed multiple nodules and/or masses, effusion or consolidation and enlarged mediastinal lymph nodes in chest CT scans than non-*Mucor* coinfection patients, it was still difficult to diagnose *Mucor* coinfection, for which the prescription of targeted treatment amphotericin B liposome is recommended (Cornely et al., 2019). Hence, the diagnosis of proven invasive pulmonary mycosis mostly depends on histopathologic, cytopathologic, or direct microscopic examination of a specimen obtained by needle aspiration or biopsy.

However, the reality is that the diagnosis of pulmonary mycosis based solely on pathology may lead to misdiagnosis and missed diagnosis. Especially when distinguishing various molds, such as *Aspergillus* and *Mucor*, confusion occurs easily because of the similarity in the morphology of these two molds. Discrimination highly depends on skill and experience. In this study, the pathological judgment of some cases also exhibited this problem. It is worth noting that for 3 patients, mixed mycosis was missed in the biopsy in the hospital to which they were first admitted. The diagnosis of mucormycosis was made after reviewing the original pathological slides from the hospitals to which they were first admitted. As stated in the EORTC/MSGERC consensus definitions of IFDs, amplification of fungal DNA by PCR combined with DNA sequencing when molds are seen in formalin-fixed paraffin-embedded tissue can be used as one of the criteria for the diagnosis of infection by molds in these situations (Donnelly et al., 2019).

Additionally, lesions in 7 patients re-enlarged after the original effective treatment and were diagnosed after a second biopsy and pathological examination. Hence, based on the comprehensive patient situation, clinicians must also have rigorous procedures in the management of patients and analyze the reasons for ineffectiveness of original treatment or re-enlargement of lesions, including the sufficient triazole concentration in blood (Di Paolo et al., 2021), drug resistance (Yousefian et al., 2021), and the possibility of mixed infection with multiple pathogens such as bacteria, tuberculosis, other fungi, etc. As shown in this study, mixed mycosis is also one of the factors that needs to be considered, even in immunocompetent patients. Hence, we recommend that the following procedures can be considered in patients with pulmonary mycosis when poor response to initial antifungal treatment happens or the initial lesions grow again, including whether treatment drug monitor shows adequate concentrations of azoles (Di Paolo et al., 2021), whether there is new occurrence of symptoms or characteristic imaging of other types fungal infections other than tuberculosis or non-tuberculosis mycobacteria, whether current in use antifungal agents could cover suspected pathogens such as fluconazole is invalid for aspergillus and voriconazole is invalid for mucor. At this time, targeted fungal etiology and pathological examination are required to confirm the diagnosis.

Except for early complete surgical treatment being strongly supported for mucormycosis whenever possible, systemic antifungal treatment is recommended as the first choice for other fungi by guidelines (Cornely et al., 2019; Garcia-Vidal et al., 2019; Groll et al., 2021). In the present study, except for one patient who died because of refusing further treatment, all mucormycosis patients recovered after receiving systemic amphotericin B treatment. One mucormycosis patient also received surgery after systemic antifungal treatment. Three cases with non-*Mucor* mixed infection received surgery after poor response to systemic antifungal treatment or for diagnosis purposes. Hence, surgery is an alternative to systemic antifungal therapy in some mixed pulmonary mycoses with a poor response. In contrast to the high mortality in immunocompromised hosts in the literature, most mixed mycosis cases in the present study had a good response.

The major limitations of our study were that it was a retrospective, single-center study. There may have a bias in patient enrollment. Another limitation was that not all patients were screened for gene mutations, such as the STAT 3 gene and the level of anti-interferon γ antibody. In the present study, only three cases with *Talaromyces marneffei* infection were tested for the STAT 3 gene and anti-interferon γ antibody, with one of them positive for each.

## Conclusion

Mixed pulmonary mycosis is not uncommon in immunocompetent hosts. During the management of mycosis, we recommend keeping mixed mycosis in mind for patients with a poor response to initial antifungal treatment, even in immunocompetent populations. A rigorous procedure may help to differentiate mixed mycosis from single mycosis.

## Data Availability Statement

The raw data supporting the conclusions of this article will be made available by the authors, without undue reservation.

## Ethics Statement

This study was approved by the ethics committee of the First Affiliated Hospital of Guangzhou Medical University (2018-119). Written informed consent for participation was not required for this study in accordance with the national legislation and the institutional requirements.

## Author Contributions

Conception and design: YZ, CL, and FY. Collection and assembly of data: YZ, CL, SL, JZ, ZL, and YG. Data analysis and interpretation: YZ. Manuscript writing: YZ, CL, and FY. Final approval of manuscript: YZ, CL, SL, JZ, ZL, YG, and FY. All authors contributed to the article and approved the submitted version.

## Funding

This research was funded by the ZHONGNANSHAN MEDICAL FOUNDATION OF GUANGDONG PROVINCE (ZNSA-2020003); Independent Fund of the State Key Laboratory of Respiratory Diseases (SKLRD-Z-202019); the Guangzhou Institute of Respiratory Health Open Project (2019GIRHZ06).

## Conflict of Interest

The authors declare that the research was conducted in the absence of any commercial or financial relationships that could be construed as a potential conflict of interest.

## Publisher’s Note

All claims expressed in this article are solely those of the authors and do not necessarily represent those of their affiliated organizations, or those of the publisher, the editors and the reviewers. Any product that may be evaluated in this article, or claim that may be made by its manufacturer, is not guaranteed or endorsed by the publisher.
